# Coelomocyte populations in the sea urchin, *Strongylocentrotus purpuratus*, undergo dynamic changes in response to immune challenge

**DOI:** 10.3389/fimmu.2022.940852

**Published:** 2022-08-31

**Authors:** Megan A. Barela Hudgell, Leon Grayfer, L. Courtney Smith

**Affiliations:** Department of Biological Sciences, George Washington University, Washington, DC, United States

**Keywords:** invertebrate, echinoderm, cellular immunity, flow cytometry, Vibrio, zymosan A

## Abstract

The sea urchin, *Strongylocentrotus purpuratus* has seven described populations of distinct coelomocytes in the coelomic fluid that are defined by morphology, size, and for some types, by known functions. Of these subtypes, the large phagocytes are thought to be key to the sea urchin cellular innate immune response. The concentration of total coelomocytes in the coelomic fluid increases in response to pathogen challenge. However, there is no quantitative analysis of how the respective coelomocyte populations change over time in response to immune challenge. Accordingly, coelomocytes collected from immunoquiescent, healthy sea urchins were evaluated by flow cytometry for responses to injury and to challenge with either heat-killed *Vibrio diazotrophicus*, zymosan A, or artificial coelomic fluid, which served as the vehicle control. Responses to the initial injury of coelomic fluid collection or to injection of *V. diazotrophicus* show significant increases in the concentration of large phagocytes, small phagocytes, and red spherule cells after one day. Responses to zymosan A show decreases in the concentration of large phagocytes and increases in the concentration of small phagocytes. In contrast, responses to injections of vehicle result in decreased concentration of large phagocytes. When these changes in coelomocytes are evaluated based on proportions rather than concentration, the respective coelomocyte proportions are generally maintained in response to injection with *V. diazotrophicus* and vehicle. However, this is not observed in response to zymosan A and this lack of correspondence between proportions and concentrations may be an outcome of clearing these large particles by the large phagocytes. Variations in coelomocyte populations are also noted for individual sea urchins evaluated at different times for their responses to immune challenge compared to the vehicle. Together, these results demonstrate that the cell populations in sea urchin immune cell populations undergo dynamic changes *in vivo* in response to distinct immune stimuli and to injury and that these changes are driven by the responses of the large phagocyte populations.

## Introduction

When Eli Metchnikoff (1) inserted a rose prickle through the body wall and into the blastocoelar cavity of a larval sea star, he demonstrated that phagocytic cells migrate to, surround, and encapsulate the foreign material. When foreign materials are injected directly into the blastocoelar cavity of larval sea urchins, they are surrounded, encapsulated, or phagocytosed by the blastocoelar cells, which mediate the larval immune system (2–4). In adults, coelomocytes clear injected particles from the coelomic cavity such as foreign cells (5, 6) and bacteria (7, 8). The clearance processes decrease the concentration of coelomocytes temporarily that is assumed to result from encapsulation or phagocytosis followed by either the degradation of the foreign material and/or the removal of phagocytes that contain the foreign material from the body of the animal (7, 8). Alternatively, injection of lipopolysaccharide (LPS) increases the concentration of coelomocytes in immunoquiescent (immuno-down regulated) sea urchins (9, 10). Thus, in response to physiological and/or immune challenge, sea urchins show a range of changes in the coelomocyte concentrations. However, no quantitative evidence is currently available for how populations of distinct coelomocyte types change in response to immune challenges. Although there are reports based on manual differential cell counts of fixed and stained phagocytes (9, 11) or live coelomocytes (12–14), several reports use flow cytometry to identify and isolate specific types of coelomocytes (15–18) but do not identify and evaluate all sea urchin coelomocyte populations.

Most of the coelomocytes are located in the coelomic fluid (CF) that fills the internal body cavity of sea urchins, while some are present in the tissues of the animals (11, 19–21) including the animal surface or cuticle (22) and on spines that are covered by epidermis with embedded coelomocytes (23–25). Coelomocytes vary in size, structure, and function (26, 27) with three major categories based on major morphological differences; phagocytes, spherule cells, and vibratile cells [reviewed in (28)]. The phagocytes are composed of four types of cells that are based on size and cytoskeletal structure. The large phagocytes are differentiated by the organization of their cytoskeleton that define their different morphologies when spread on glass; discoidal phagocytes are disc shaped whereas the polygonal phagocytes are polygon shaped (9, 29–31). Medium phagocytes are intermediate in size and show an unusual pentagonal or hexagonal shape when spread on glass and appear when 1 mL of CF is depleted experimentally (11). Small phagocytes typically have filopodial morphology when spread on glass (11, 32, 33) but show a different morphology in suspension (34). Although the phagocyte categories are based on size and morphology, and subsets of each type may have functional differences based on differences in protein expression and secretion such as the complement homologue, SpC3 (32, 33, 35), and the SpTransformer proteins (9, 36). The spherule cells have large cytoplasmic spherules or vesicles that are either red or colorless (26, 37, 38). Red spherule cells (RSCs) contain the red pigment, echinochrome A, in their cytoplasmic vesicles. Colorless spherule cells (CSCs) are of similar size and morphology as the RSCs, but do not produce red or other colored pigments. Vibratile cells are spherical with a single long flagellum [reviewed in (27, 28)]. All seven of these coelomocyte types are present in the CF in *S. purpuratus*, which can be collected easily for evaluation without sacrificing the animal. For images of these cell types, see Box 1.2 in (39).

The quantification of coelomocyte populations has generally relied on microscopy and differential cell counts (9, 11, 21, 30). However, analyses of coelomocyte populations in echinoids have often been restricted to the phagocyte populations because they bind tightly to glass slides and have ignored other populations of coelomocytes because they do not bind glass (7–9, 20, 30, 40, 41). An improvement to this approach has been to use flow cytometry, and although it has been reported previously for coelomocytes from *S. purpuratus*, identification of specific cell types were not reported (34, 42). To improve the analysis and quantification of sea urchin coelomocytes and to circumvent problems of cell binding (or not binding) to slides followed by manual cell counts, we reported the identification of different cell populations by differential flow cytometry gating strategies. Gates were established and optimized by evaluating fractions of coelomocyte types separated by density centrifugation to identify the large phagocytes that are a mixture of discoidal and polygonal phagocytes, small phagocytes, RSCs, and a mixture of vibratile cells and CSCs (43). By using these gates, accurate quantification of changes in coelomocyte populations can be explored in response to various challenges. Here, this flow cytometry approach is used to track, quantify, and analyze the response of coelomocyte populations over time for individual sea urchins. We show that coelomocyte populations change in animals responding to a puncture injury that is combined with a withdrawal of CF, to challenge with a Gram negative marine bacterial species, *Vibrio diazotrophicus*, or to zymosan A, compared to injuries incurred from the injection from the vehicle control (artificial coelomic fluid, aCF). We show that the cellular responses to small *Vibrio* cells are different from responses to large zymosan A particles. The overall results suggest that the coordination of the cellular immune response of the sea urchin appears to be based on the activities and population changes of the large phagocytes, which make up 65-70% of the coelomocytes (6, 44) with responses from RSCs and small phagocytes occurring in the event that large phagocytes may not clear foreign particles efficiently.

## Materials and methods

### Sea urchin care

Sea urchins were collected by and purchased from Marinus Scientific (Long Beach CA), or they were collected from the near shore of the Pacific Ocean near San Diego CA and purchased from the Southern California Sea Urchin Company (Corona del Mar CA). Animals were maintained as described (33). Briefly, they were fed re-hydrated kelp (*Saccharine japonica*; Wel-Pac Dashi Kombu), housed for at least two years in marine aquaria, and were assumed to be immunoquiescent (10, 45) prior to the start of the study.

### Coelomocyte collection

CF (200 μL) was withdrawn from sea urchins according to (42, 43). This sampling approach evaluates the coelomocytes that are in the coelomic fluid, which does not include coelomocytes in peripheral tissues. These volumes were, at most, 0.83% to 1.17% of the total volume of CF in adult sea urchins, which was estimated based on weight according to (46). Briefly, cellular clotting was prevented by the addition of ice cold calcium- and magnesium-free sea water with EDTA and HEPES (CMFSW-EH; 460 mM NaCl, 10.73 mM KCL, 7.04 mM Na2SO_4_, 2.38 mM NaHCO_3_, 70 mM EDTA, 20 mM HEPES pH 7.4) that was at least equal in volume to the collected CF (36). The diluted CF was held on ice for up to one hour prior to further use. Coelomocyte concentration was estimated for individual sea urchins prior to CF collection and dilution according to previous reports (36, 43).

### Preparation of foreign particles

*Vibrio diazotrophicus* (American Type Culture Collection; item #33466) (47) was grown in marine broth (Difco Laboratories) as described (43, 48, 49). *Vibrio* cells were re-suspended in artificial CF (aCF; 10 mM CaCl_2,_ 14 mM KCL, 50 mM MgCl_2_, 390 mM NaCl, 1.7 mM Na_2_HCO_3,_ 25 mM Na_2_SO_4_ (45), adjusted to 10^4^ and 10^6^ bacteria/µL in 1 mL aliquots, heat-killed and stored at -20°C.

Zymosan A particles (1 mg) from *Saccharomyces cerevisiae* (Sigma-Aldrich: Product number Z4250 CAS#58856-93-2) were rehydrated in 100 μL of aCF, heated to 100°C in a water bath for 1 hour and centrifuged at 1700 x *g* for 30 minutes according to the supplier’s protocol. The pellet was resuspended in aCF at a final concentration of 2 x 10^5^ particles/μL and stored at -20°C.

### Immune stimulation

Sea urchins were injected initially with 10^4^ heat-killed *V. diazotrophicus* per ml of CF on day 2 and 10^6^ V*. diazotrophus* per ml of CF on day 5 as reported (43), or with zymosan A, or vehicle (aCF) by injection through the peristomial membrane as described (42). The number of *V. diazotrophicus* cells/ml or zymosan A particles/mL that were injected was based on the estimated volume of the CF for each animal according to (50). The optimal number of zymosan A particles injected per mL of CF was determined by comparing the surface area of a *V. diazotrophicus* cell (~3.5 μm^2^) as calculated using the surface area of a rod (47) compared to the surface area of a zymosan A particle (~28 μm^2^) (51). Preliminary experiments to evaluate adverse reactions, such as changes in behavior and appearance, indicated that the initial injection of about 2 x 10^3^ zymosan A particles/mL of CF was deemed acceptable for analysis of coelomocytes. This dosage presented a somewhat similar surface area compared to 10^4^ heat-killed *V. diazotrophicus*/ml of CF, as previously described (52). For the second injection, the same test animal received 2 x 10^5^ zymosan A particles/mL of CF that was based on similar surface area as 10^6^ V*. diazotrophicus*/ml CF. However, because of adverse animal reactions, the concentration of zymosan A particles for the second injection was reduced to 2 x 10^4^ particles/mL of CF.

Baseline levels of coelomocytes were established by an initial collection of CF (200 μL; n = 18 samples) from sea urchins (n = 11; see **Table S1**) on day 0, which was followed by CF collection on day 1 that evaluated responses to the injury on day 0. On day 2, sea urchins (n = 8) were injected with 30 µL of heat-killed *V. diazotrophicus* that resulted in 10^4^ bacteria per mL of CF, and on day 5 they received an additional injection of 30 µL that resulted in 10^6^ bacteria/mL of CF (16, 48). Control animals (n = 7) received injections of 30 µL vehicle on days 2 and 5. On days 3 and 6, CF (200 μL) was collected for analysis of coelomocyte populations by flow cytometry. In some cases, the same sea urchin was used to analyze responses to both vehicle and either *V. diazotrophicus* or zymosan A, which were carried out two to three months apart.

Some sea urchins were used as their own controls. For those animals that received injections of *V. diazotrophicus* (n=4), three (SU-V2, SU-V3, SU-V4) received injections of vehicle followed by a month of recovery before challenge with *V. diazotrophicus*. One sea urchin (SU-V1) was treated in the opposite order; injected initially with *V. diazotrophicus* followed by five months of recovery before injection with vehicle. For the sea urchins that received injections of zymosan A (n=3), they received injections of vehicle followed by a month of recovery before challenge with zymosan A.

### Flow cytometry

Analysis and quantification of live coelomocytes by flow cytometry was conducted according to Barela Hudgell et al. (43). Briefly, gates were established using Percoll density gradient centrifugation to separate coelomocyte populations into fractions as described (42, 46). Each fraction was evaluated separately by flow cytometry to establish gates for large phagocytes, small phagocytes, RSCs, and a mixture of CSCs and vibratile cells (**Figure S1**). Preliminary gating was used to gate for total coelomocytes as reported by (42). Here, the term ‘total coelomocytes’ refers to the flow cytometry gate that encompassed all live coelomocytes in a sample.

### Fold change calculation

The fold change for each sampling time point was calculated relative to baseline data (day 0), which was set to 1. Combined fold changes were calculated to quantify population differences either over time or between experimental sets and were based on the numerical difference between two data points.

### Statistical analysis

Two-tailed, unequal variance, unpaired *t*-tests, and one-way ANOVA were carried out in Excel (Microsoft) and used to determine significant differences among groups. Both the Quartile and τ tests were used to identify outlier data from two animals that were omitted from further analysis. Paired *t*-tests were used to compare data from the same animal responding at different times to the challenge with foreign particles compared to responses to the vehicle control. Significance was set at *p* ≤ 0.05 for all ANOVA and *t*-tests.

## Results

### Coelomocyte concentration decreases in response to a single puncture injury

**Figure 1:**
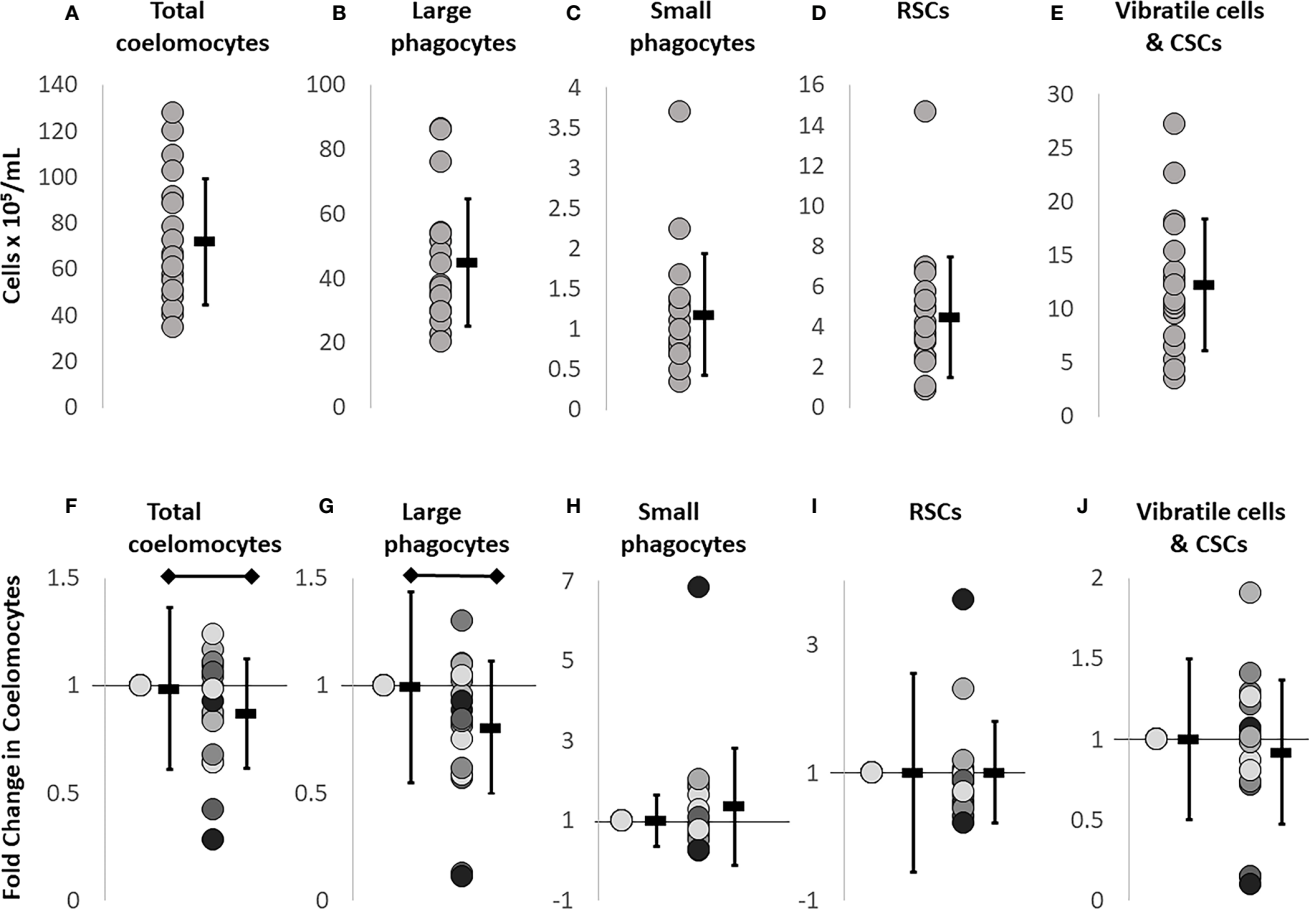
Baseline coelomocyte concentrations in sea urchins prior to challenge are used as the reference for subsequent treatments. CF samples collected on day 0 (n = 18 samples) were evaluated for the different types of coelomocytes at baseline, prior to any experimental manipulations, and were used for comparison to all other time points. Each dot represents the result for an individual sea urchin. **(A)** Total coelomocyte concentrations are determined by cell counts with a TC20 automatic cell counter (BioRad). **(B–E)** Different types of coelomocytes are evaluated by flow cytometry (Accuri C6 Flow Cytometer, BD Biosciences) that identifies and quantifies concentrations of different coelomocyte populations at baseline. **(F)** Injury and CF withdrawal induces a fold decrease in large phagocytes compared to baseline (day 0). **(G–J)** Different types of coelomocytes from sea urchins (n = 11) are re-evaluated on day 1 to characterize the response to the puncture injury and CF withdrawal carried out on day 0. To calculate fold changes on day 1, baseline data shown in panels **(A–E)** are set to 1 in panels **(F–J)**. The average ± SD is shown to the right of each data set. The black horizontal bars indicate significant differences (unpaired *t*-test; *p* ≤ 0.5). Note that the Y axes are not the same in the different panels.

CF withdrawal or injections into the coelomic cavity, may incur more damage to sea urchins than just to the surface skin. The needle punctures the peristomial membrane, passes through the peripharyngeal cavity, and punctures the peripharyngeal membrane to access the CF in the coelomic cavity. This may also damage a radial nerve located on the inside surface of the peristomial membrane [for echinoid internal anatomy, see (19)]. To determine whether there was a response to this puncture injury, cells from CF withdrawals on day 0 and day 1 were compared by flow cytometry. The baseline data for coelomocyte concentration established on day 0 differed among sea urchins (n = 11) (**Figures 1A–E**). The injury response evaluated on day 1 was calculated as a fold change difference from the baseline values, to discern changes due to injury responses (**Figures 1F–J**). Total coelomocyte numbers showed a modest but significant fold decrease (*p* = 0.049) after injury (day 1) compared to baseline (day 0, **Figure 1F**). The basis for this change was a significant fold decrease (*p* = 0.0085) in the large phagocytes (**Figure 1G**), whereas no significant changes were noted for the other cell types (**Figures 1H–J**). This indicated that the injury response primarily altered the large phagocyte population, which was the basis for changes in total coelomocytes.

### Sea urchin coelomocytes show fold increases following challenge with *Vibrio diazotrophicus*

**Figure 2:**
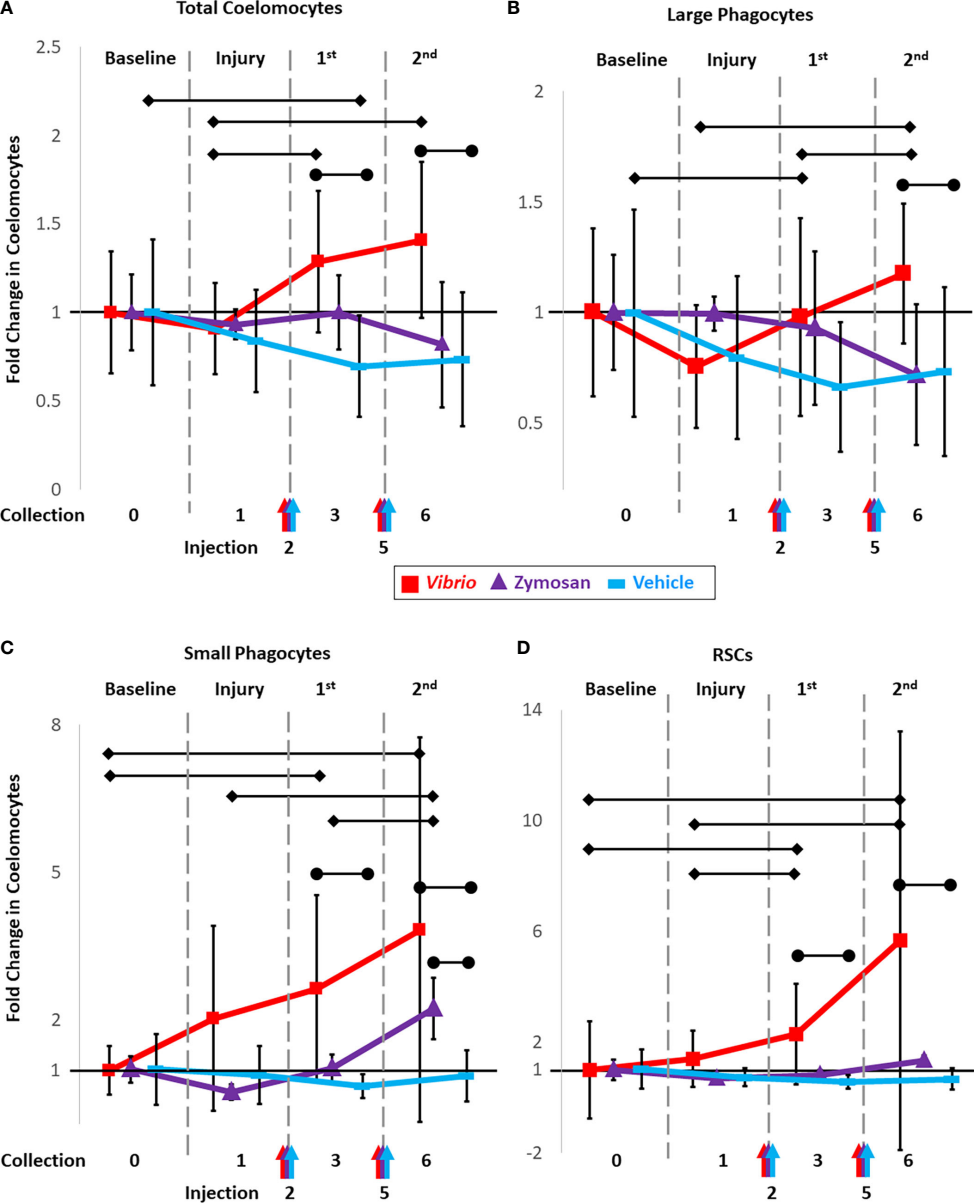
Injections of heat-killed *Vibrio diazotrophicus*, zymosan A, or vehicle (aCF) induce fold changes in total coelomocytes and in populations of different coelomocytes. Sea urchins were injected on days 2 and 5 (arrows along the x-axis) with either *Vibrio diazotrophicus* (n = 8; red), zymosan A (n = 3; purple), or vehicle as the injury control (n = 7; blue) and samples for analysis of fold changes were collected on days 3 and 5. Total coelomocytes **(A)** along with populations of large phagocytes **(B)**, small phagocytes **(C)**, and RSCs **(D)** were analyzed by flow cytometry and tracked over time. Responses to challenges are compared to both baseline (day 0; set to 1) and to injury and CF withdrawal (day 1). Black vertical lines indicate the ± SD for each time point for the three experimental groups. See **Figures S2-S4** for graphs showing the responses to the two challenges and the vehicle control for total coelomocytes and for the different coelomocyte populations from individual sea urchins over time. Black horizontal bars with diamond ends indicate significant differences between days (unpaired *t*-test; significance = *p* ≤ 0.05). Black horizontal bars with circle ends indicate significant differences between experimental groups vs. the vehicle group (ANOVA; significance set = *p* ≤ 0.05.).

To determine whether injections of foreign particles altered coelomocyte concentrations, sea urchins were injected on day 2 of the experimental protocol with either *Vibrio diazotrophicus* (n = 8 animals), zymosan A (n = 3 animals), or vehicle (n = 7 animals; **Table S1**). CF was collected on day 3 and evaluated for fold changes in total coelomocytes and for changes in the different cell populations relative to both baseline and to injury. Fold changes in total coelomocytes in response to *V. diazotrophicus* were not different from baseline but were increased compared to the injury response (*p* = 0.018) (**Figures 2A**, red line; **S2A-C**). Sea urchins injected with zymosan A showed no changes in total coelomocytes on day 3 compared to either baseline or injury (**Figure 2A** purple line), which was due to the variability of the responses and the small sample size (**Figure S2B**). Sea urchins injected with vehicle alone showed a significant fold decrease in coelomocytes on day 3 compared to baseline (*p* = 0.04), but there was no fold difference in total coelomocytes compared to injury from CF collection (**Figures 2A**, blue line; **S2C**). Although there was variability among animals in the three groups (**Figures S2A-C**), the results suggested differences in coelomocyte responses to different types of foreign particles and to vehicle.

The same groups of sea urchins were injected again on day 5 with either an increased number of *V. diazotrophicus* cells, an increased number of zymosan A particles, or vehicle (see methods). Two injections were employed in this analysis based on previous reports showing that two or more injections of immune stimulators resulted in a more robust immune response in sea urchins (16, 45, 48, 53). CF was collected from all animals on day 6 and coelomocytes were evaluated. In general, there was a significant fold increase (*p =* 0.018) in total coelomocytes after the second injection of *V. diazotrophicus* compared to injury (**Figures 2A** red line; **S2A**). However, the response to the second injection of *V. diazotrophicus* was not different from the response to the first injection, nor was it different from baseline. Sea urchins injected the second time with zymosan A showed no significant fold changes in coelomocytes compared to baseline (**Figures 2A**, purple line; **S2B**), although responses to the first injection of vehicle resulted in a significant fold decrease in coelomocytes compared to baseline (**Figures 2A**, blue line; **S2C**). When results from days 3 and 6 were compared among groups receiving different challenges, significant differences in fold changes (*p* ≤ 0.05) were noted for coelomocytes in sea urchins injected with *V. diazotrophicus* compared to vehicle, whereas there were no differences in coelomocytes from sea urchins injected with zymosan A compared to vehicle (**Figures 2A**; **S3A**). Overall, injections of both *V. diazotrophicus* and vehicle induced fold changes in coelomocytes while zymosan A did not. However, variations in coelomocytes among animals within and among groups was evident and tended to confound statistical analyses to identify any additional significant changes.

### Different populations of coelomocytes show dynamic changes in response to immune stimuli

**Figure 3:**
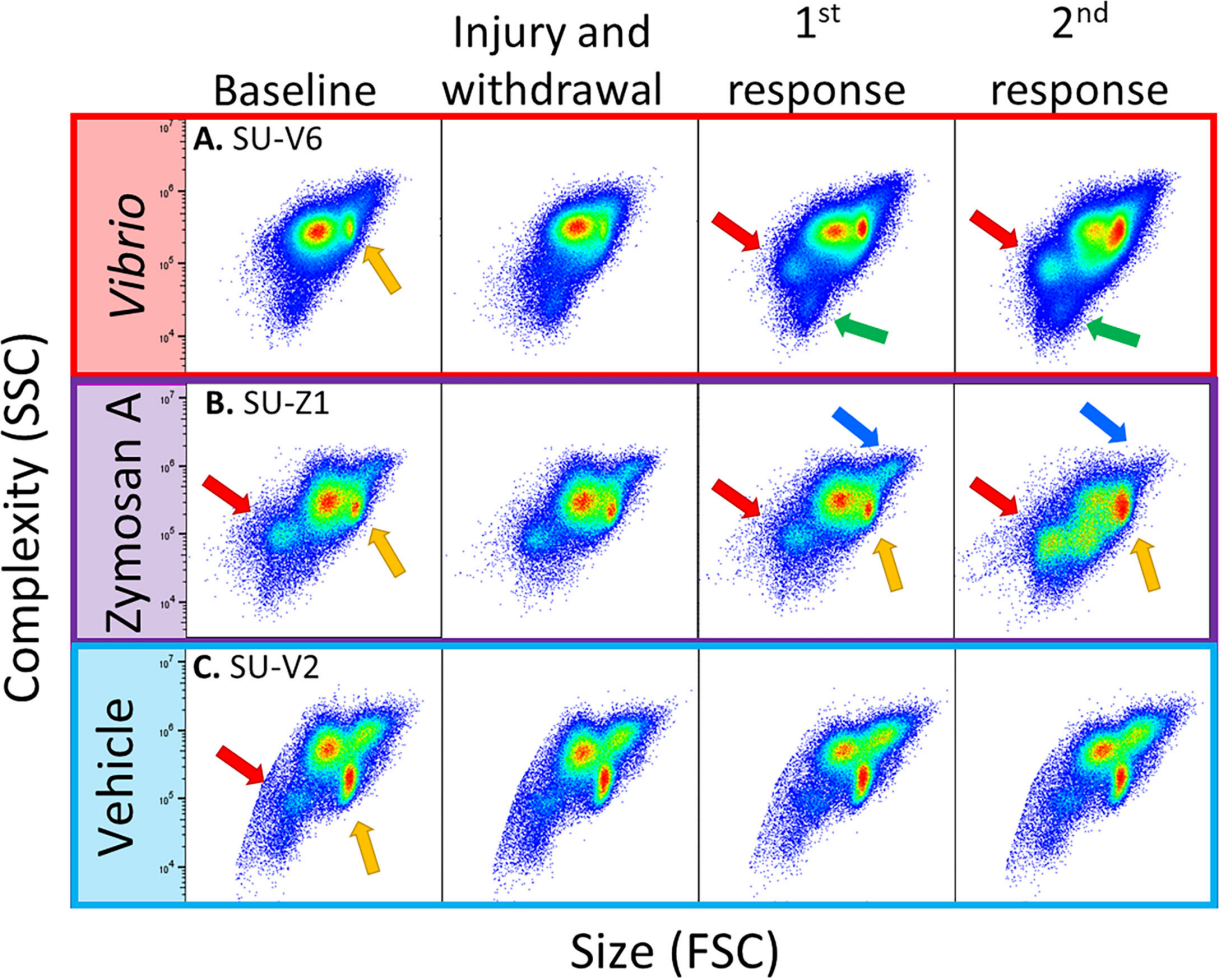
Flow cytometry illustrates diversity of coelomocyte composition among sea urchins responding to immune challenge. Scatter plots of selected sea urchins **(A–C)** from each experimental group shows responses to *Vibrio diazotrophicus* (red), zymosan A (purple), or vehicle (blue). The plots show the gate for total coelomocytes to illustrate differences and changes in coelomocyte populations among animals. Coelomocytes were evaluated by flow cytometry at baseline (day 0), after injury and CF withdrawal (day 1), and 24 hours after the first and second response to immune challenge or vehicle (days 3, 6). Different coelomocyte populations are indicated by arrows: large phagocytes (blue), RSCs (red), vibratile and CSCs (yellow), and small phagocytes (green). See **Figure S1** that shows different populations of coelomocytes as identified by flow cytometry.

Flow cytometry scatter plots from animals challenged with foreign particles illustrated dynamic changes in coelomocyte populations (**Figure 3**). Variations among the coelomocyte populations were observed among different animals at both baseline and in response to the initial injury and CF withdrawal. Variations at baseline were most clearly demonstrated by the mixed population of vibratile cells and CSCs that could be discerned in all three animals prior to any experimental stimulation or injury (**Figure 3**, yellow arrows). Sea urchins B and C had discernable populations of RSCs at baseline (**Figure 3**, red arrows). Responses to the initial injury and CF withdrawal did not show any major changes to the coelomocyte populations compared to baseline for animals. Responses to the foreign particles resulted in a range of changes in cell populations among the animals, including increases in specific populations or a shift in their location on the scatter plots. Sea urchins A and B showed, an increase in the different populations of coelomocytes in response to *V. diazotrophicus* and zymosan A including the RSCs (red arrows) and the small phagocytes (green arrows) as indicated by the shift in color on the scatter plot from blue to green (**Figure 3**). In sea urchin B challenged with zymosan A, the mixed population of vibratile cells and CSCs also expanded (**Figure 3**, yellow arrows). Sea urchin B also showed dynamic and variable changes in the large phagocytes with an expansion of the region on the scatter plot for large phagocytes (**Figure 3**, blue arrows). This may be indicative of phagocytosis of the zymosan A particles that would be detected as an increase in the complexity of some large phagocytes. Sea urchin C that received vehicle injections did not show any notable changes in cell populations (**Figure 3**). In general, these results indicated that flow cytometry could be used to identify changes over time in different coelomocyte populations among animals responding to different challenges.

To understand these dynamic changes that coelomocyte populations appear to undergo in response to foreign particles, CF samples were evaluated using flow cytometry gates established in (43) to characterize responses of different types of coelomocytes to the initial injury and CF withdrawal, and to injections of *V. diazotrophicus*, zymosan A, or vehicle. Changes were tracked and reported as fold increases or decreases for each coelomocyte population relative to their baseline composition, which was set to 1 (**Figures 2B–D**; **S2D-L**; **S4A-C**). Although the large phagocyte population did not show a significant change in response to the first injection of *V. diazotrophicus* compared to injury, the second injection resulted in a significant fold increase compared to both the first injection of *V. diazotrophicus* (*p* = 0.05) and injury (*p* = 0.001) (**Figures 2B**, red line; **S2D**). Fold changes in the large phagocyte population in response to both injections of zymosan A were highly variable among animals and therefore showed no significant changes relative to baseline or injury (**Figures 2B**, purple line; **S2E**). For sea urchins that received injections of vehicle, the large phagocyte population showed a fold decrease in response to injury, which remained decreased in response to the two injections of vehicle including a significant fold decrease (*p* = 0.03) after the first injection of vehicle compared to baseline (**Figures 2B**, blue line; **S2F**). In general, the large phagocyte population showed variable changes among animals in response to injections of *V. diazotrophicus* or zymosan A with a decreased fold change in response to injections of vehicle.

The small phagocyte population responded to both injections of *V. diazotrophicus* with a fold increase (*p =* 0.03 and *p* = 0.05, respectively) relative to baseline (**Figures 2C**, red line; **S2G**). However, these changes in the small phagocytes were not different from responses to injury due to the variability of the response in one animal (**Figure S2G**). The second injection of zymosan A resulted in a significant fold increase in small phagocytes compared to injury (*p* = 0.03), and the first injection of zymosan A (*p* = 0.02; **Figures 2C**, purple line; **S2H**). Changes in the small phagocyte population in response to injections of vehicle were highly variable and showed no fold changes relative to baseline or injury (**Figures 2C**, blue line; **S2I**). In general, for many of the sea urchins, injections of both *V. diazotrophicus* and zymosan A induced fold increases in small phagocytes. The RSC population showed significant increases in response to the first (*p* = 0.01) and second injections (*p* = 0.03) of *V. diazotrophicus* compared to baseline and to injury, respectively (**Figures 2D**, red line; **S2J**). While there was a distinct pattern of fold increases in RSCs in response to zymosan A, particularly for the second injection, these changes were not significant (**Figures 2D**, purple line; **S2K**). Sea urchins injected with vehicle showed great variability in the population of RSCs (**Figures 2D**, blue line; **S2L**). Based on these results and the variability among sea urchins, significant fold changes in the population of RSCs were only detected in response to injection with *V. diazotrophicus*. The mixed population of vibratile cells and CSCs showed highly variable changes among animals in response to injections of both foreign particles and the vehicle however none of these changes were significant (**Figure S4A-C**). Overall, fold changes in the population of vibratile cells and CSCs were highly variable among sea urchins, showing little correlation to challenges with either of the foreign particles or to the vehicle control.

### Coelomocyte populations shift following immune challenges

To parse out whether coelomocyte populations were responding to injections of vehicle (injury) and/or to injections of foreign particles suspended in the vehicle, comparisons were made among groups. Sea urchins that received either *V. diazotrophicus* or zymosan A were compared with those that received only vehicle. Responses to challenges were first compared to baseline to define fold changes in different coelomocyte populations. Next, combined fold changes were calculated as a comparison between responses to the foreign particles and the response to vehicle (**Figures 2B–D**; **S3B-D**). The combined fold change response to *V. diazotrophicus* compared to that for vehicle was significant for most of the coelomocyte types. Large phagocyte populations were significantly different in response to the second injection with *V. diazotrophicus* compared to vehicle (*p* = 0.01) (**Figures 2B**, red vs. blue lines; **S3B**). The population of small phagocytes in sea urchins responding to the first injection and second injections of *V. diazotrophicus* showed a significant combined fold change compared to vehicle (*p* = 0.02 and *p* = 0.018, respectively) that increased from 2-fold to 3-fold change, respectively (**Figures 2C**, red vs. blue lines; **S3C**). The population of RSCs showed a combined fold change of 1.8 in sea urchins responding to the first injection of *V. diazotrophicus* compared to the first injection of vehicle (*p =* 0.008) and a combined fold change of 5 to the second injection of *V. diazotrophicus* compared to vehicle (*p =* 0.005) (**Figures 2D**, red vs. blue lines; **S3D**). The only population that showed no significant changes was the mixed population of vibratile cells and CSCs (**Figure S4D**). In response to zymosan A, the fold changes in the populations of large phagocytes, RSCs, and the mixed population of vibratile cells and CSCs were essentially the same as responses to vehicle (**Figures 2B, D**, purple vs. blue lines; **S3B**, **D**; **S4D**). Only the small phagocyte population showed a significant combined fold change in response to the second injection of zymosan A compared to vehicle (*p* = 0.01) (**Figures 2C**; **S3C**). Overall, sea urchins responded to *V. diazotrophicus* with increases in the large phagocyte population that was not observed for responses to zymosan A. Both *V. diazotrophicus* and zymosan A induced increases in the populations of small phagocytes compared to responses to vehicle, although the response to zymosan A appeared delayed relative to that for *V. diazotrophicus*. Responses to *V. diazotrophicus* resulted in increased populations of large and small phagocytes and RSCs, while zymosan A only induced significant increases in the populations of small phagocytes.

### Immune stimuli alter coelomocyte proportions

**Figure 4:**
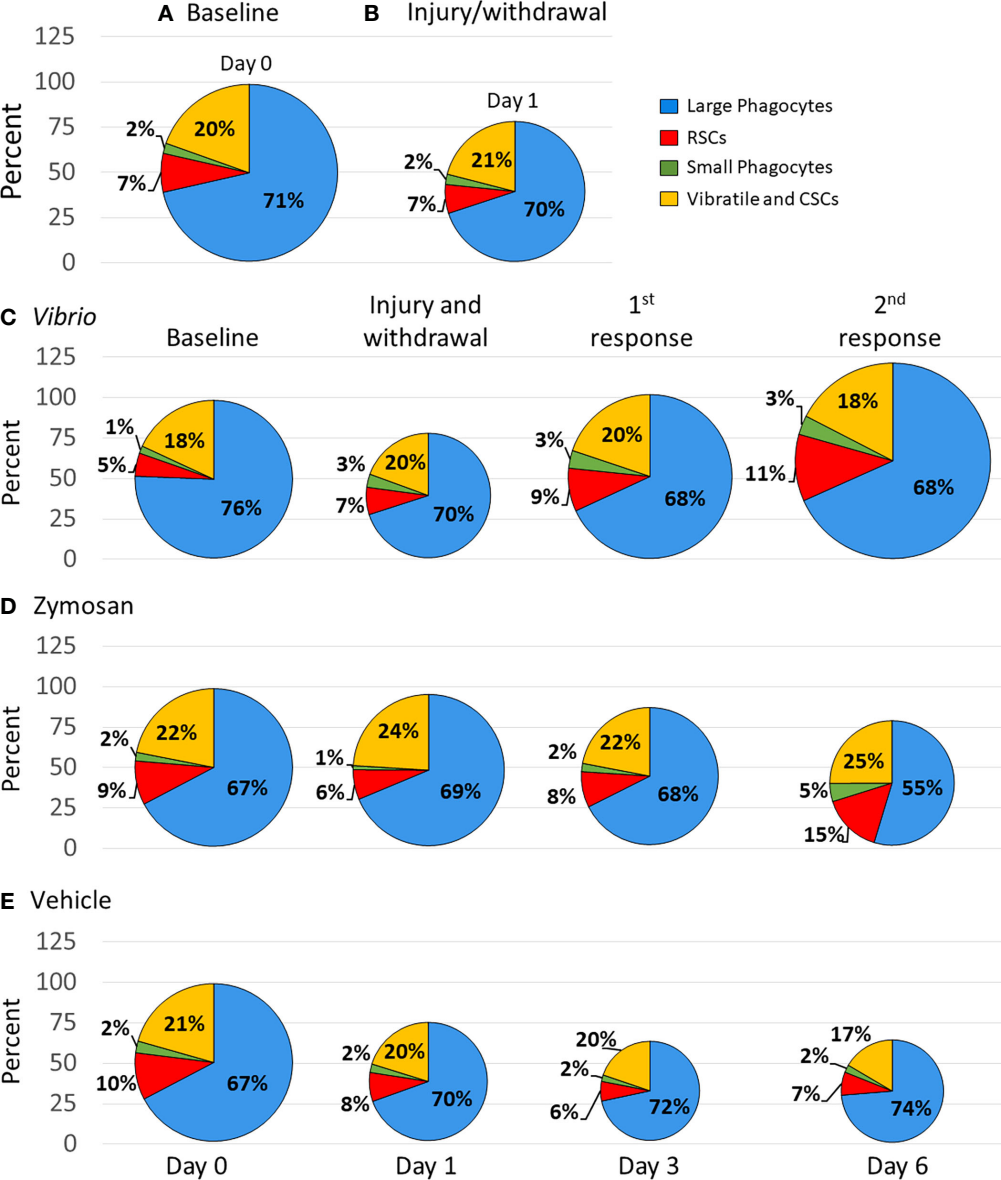
Injection of foreign particles shifts the proportions of coelomocyte types over time. Pie charts indicate the average proportions, shown as percentages, for each coelomocyte type calculated from the coelomocyte concentrations displayed in **Figure 1**. The size of the pie charts are scaled according to the average concentration of coelomocytes at each time point, with baseline (day 0) standardized to 100%. Percent changes at each time point can be inferred from the different sizes of the pies, which are indicated by the size of each pie and by the y axes. Results are shown for all sea urchins in all groups at base line **(A)** and after initial injury and withdrawal of CF **(B).** The average coelomocyte proportions are shown for each experimental group that received injections of either *Vibrio diazotrophicus* **(C),** zymosan A **(D),** or vehicle **(E)**.

While fold changes were used to evaluate variations in individual coelomocyte populations over time (**Figure 2**), these results did not address the question of the relative proportions of these cell types and how changes in one population may have influenced changes in the populations of other cell types. For example, if the population for one cell type decreased, this decrease may be accounted for by a decrease in that specific population, increases in other cell population(s), or all populations decreasing relative to the cell type in question. To address this, each cell population was re-evaluated as a proportion of total coelomocytes and reported as percentages to determine how each population changed relative to the others. Total coelomocyte concentrations (**Figure 1A**) were displayed as pie charts and the size of the pies for baseline data were normalized to 100% for all groups (**Figure 4**). Differences in the pie sizes at subsequent time points corresponded with changes in total coelomocyte concentration as more or less than 100%.

The initial analyses employed data from all sea urchins (n = 11; **Table S1**) because all animals were evaluated at baseline that required a needle puncture injury and CF withdrawal followed by the evaluation of the first sample collection that required a second needle puncture injury and CF withdrawal. In response to injury, there was a decrease in total coelomocytes compared to baseline, but the proportions of the coelomocyte populations changed very little (**Figures 4A, B**). The group of animals challenged with *V. diazotrophicus* (n = 8; **Table S1**) showed a decrease in the proportion of total coelomocytes in response to injury (**Figure 4C**). However, the first injection of *V. diazotrophicus* resulted in the proportion of total coelomocytes returning to about baseline level and increasing above baseline levels in response to the second injection. When the proportions of individual coelomocyte populations were evaluated in response to *V. diazotrophicus*, the large phagocyte proportion decreased compared to injury and baseline (**Figure 4C**, blue sectors), while the small phagocyte proportion increased post injury relative to baseline but remained unchanged after injection with *V. diazotrophicus* (**Figure 4C**, green sectors). The RSC population showed the greatest increase in proportion compared to the other cell types, with progressive increases at each sampling day in response to injury and to both injections of *V. diazotrophicus* (**Figure 4C**, red sectors). The vibratile cells and CSCs showed ±2% variation in proportions that did not correlate with responses to injury or the two injections of *V. diazotrophicus* (**Figure 4C**, yellow sectors). The major changes in cell populations was a proportional decrease in large phagocytes in response to injury and an increase in RSCs in response to *V. diazotrophicus* in addition to associated dynamic shifts in all other coelomocyte populations.

The group of sea urchins that received zymosan A (n = 3, **Table S1**) showed a decrease in the proportion of total coelomocytes after injury compared to baseline and an even greater decrease in response to the second injection of zymosan A relative to injury (**Figure 4D**). Over the course of injections of zymosan A, the proportion of large phagocytes decreased, and the proportion of small phagocytes increased compared to baseline. The proportion of the RSCs increased in response to the second injection of zymosan A compared to the previous sampling days, while the populations of vibratile cells and CSCs showed little change in proportions in response to zymosan A, which was similar to the response to *V. diazotrophicus*. The major change in the coelomocytes in response to the second injection with zymosan A was a decrease in the large phagocytes and an increase in the RSCs.

The control group that received injections of vehicle (n = 7, **Table S1**) resulted in progressive decreases in the proportion of total coelomocytes from day 1 to day 6 compared to baseline (**Figure 4E**). There were minor changes in the proportions of the coelomocyte types over the course of injury and injections with vehicle (**Figure 4E**). The proportions of large phagocyte showed minor increases, the small phagocytes did not change in response to injury or to the vehicle, the RSCs were variable but similar across responses to injury and both injections of vehicle, and the vibratile cells and CSCs decreased in response to the second injection of vehicle relative to baseline. Although injections of vehicle did not alter the relative proportions of the different coelomocyte populations, this response to repeated injuries showed a decrease in total coelomocytes. Overall, this analysis of proportions illustrated that coelomocytes responded to injection of foreign particles with a range of changes in relative proportions that were variable but appeared driven by the type or size of the foreign particle that was injected. Furthermore, changes in proportions of coelomocyte populations in response to particles were distinct from responses to vehicle. *V. diazotrophicus* induced an increase in total coelomocytes in which the relative proportions of different coelomocyte populations remained the same, while zymosan A induced a decrease in total coelomocytes with an associated dynamic shift in all coelomocyte proportions. These responses were quite different from the responses to vehicle in which there was a decrease in total coelomocytes but changes to the relative proportions of different coelomocyte populations were generally not observed.

### Sea urchins that serve as their own controls also show dynamic changes in coelomocyte populations in response to foreign particles

**Figure 5:**
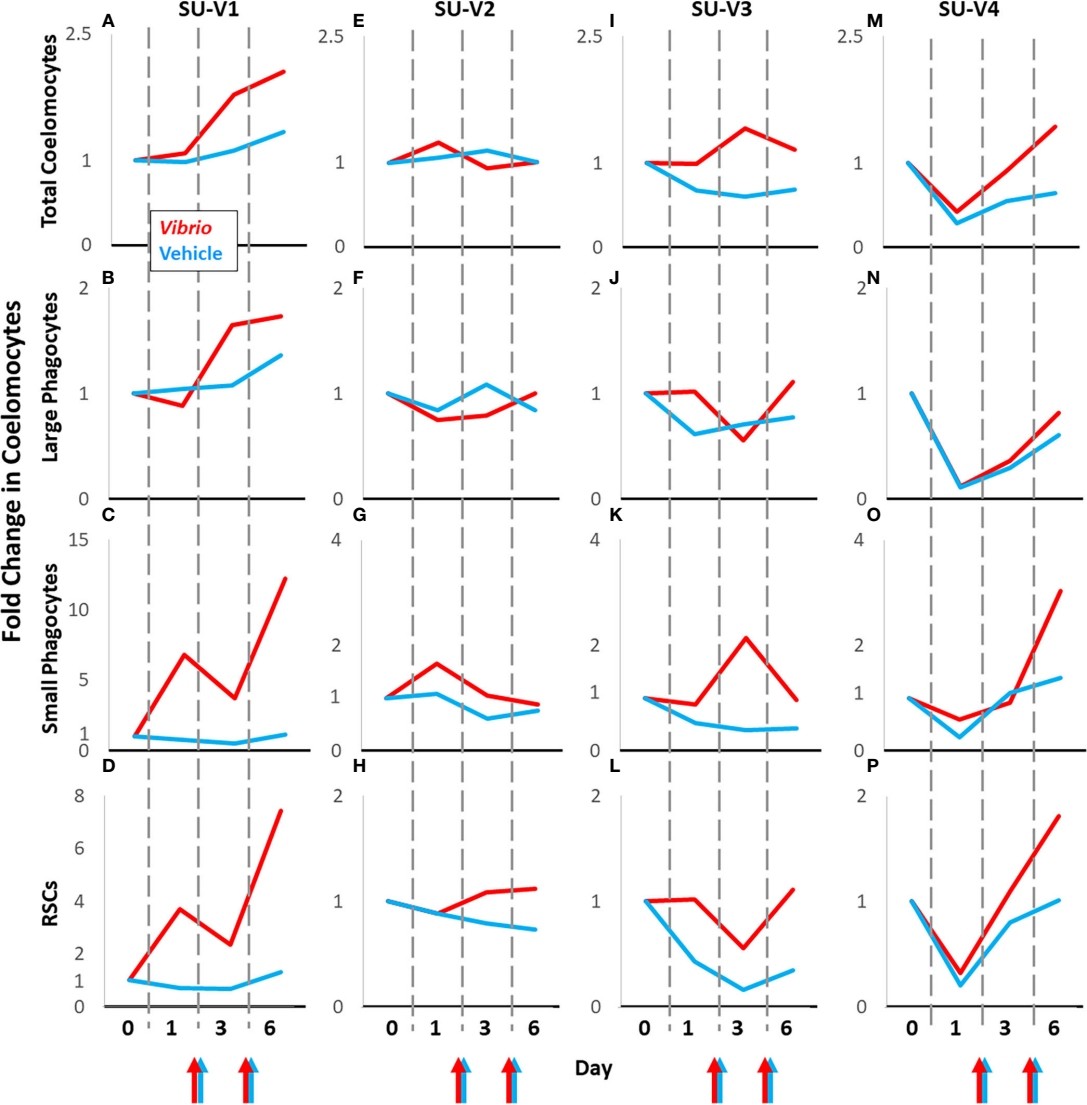
Coelomocyte responses to injection with *Vibrio diazotrophicus* is variable among sea urchins. Coelomocytes from sea urchins [SU-V2 **(E–H)**, SU-V3 **(I–L)**, SU-V4 **(M–P)**] were evaluated initially for fold changes after two injections of vehicle (blue lines). After recovery, they were injected twice with *Vibrio diazotrophicus* and fold changes in coelomocytes were evaluated again (red lines). Colored arrows at the x axes indicate days 2 and 5 when sea urchins were injected with either *Vibrio diazotrophicus* (red arrows) or vehicle (blue arrows). SU-V1 **(A–D)** was treated in the opposite order; *V. diazotrophicus* first, and then vehicle after recovery. All fold changes are relative to baseline (day 0, set to 1). Fold changes in coelomocytes are compared across days for each animal. See **Figures S5A-D** for fold changes in the mixed population of vibratile cells and CSCs in individual sea urchins responding to *V. diazotrophicus* compared to vehicle.

**Figure 6:**
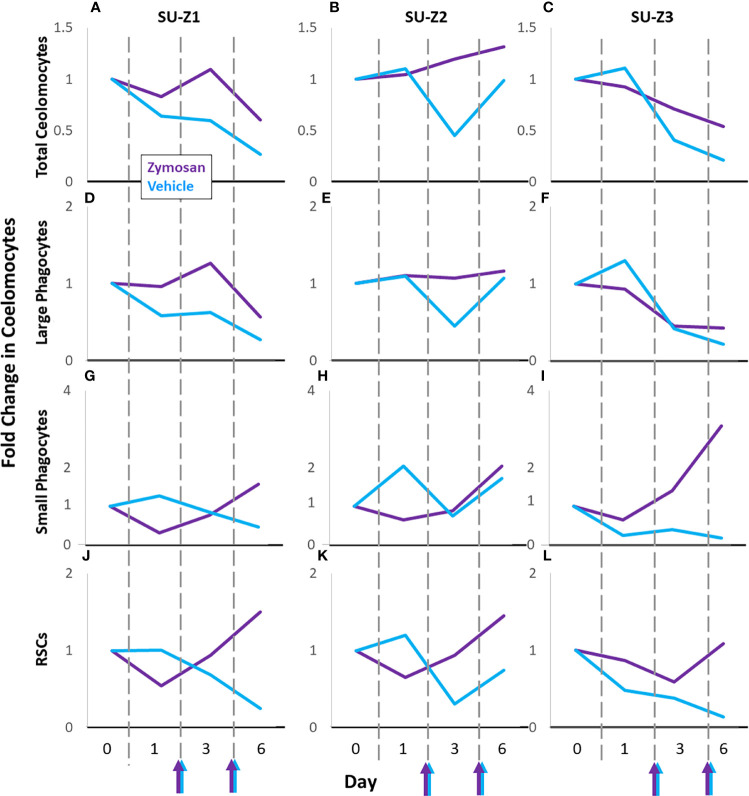
Coelomocyte responses to injection with zymosan A are variable among sea urchins. Different types of coelomocytes from sea urchins [n = 3; SU-Z1 **(A, D, G, J)**, SU-Z2 **(B, E, H, K)**, SU-Z3 **(C, F, I, L)**] were evaluated initially after two injections of vehicle (blue lines). After a month of recovery, sea urchins were injected twice with zymosan A and fold changes in coelomocyte types were evaluated again (purple lines). Colored arrows at the x axes indicate days 2 and 5 when sea urchins were injected with either zymosan A (purple arrows) or vehicle (blue arrows). All fold changes are relative to baseline (day 0, set to 1). Fold changes in the different types of coelomocytes are compared across days for each animal. See **Figures S5E-G** for fold changes in the mixed population of vibratile cells and CSCs in individual sea urchins responding to zymosan A compared to vehicle.

The status of prior contact with pathogens and microbes in sea urchins is unknown during housing in recirculating aquaria and before coelomocyte collection for this study. Their immunological responsiveness is complicated by the genetic diversity among sea urchins (54). Consequently, at least these two factors result in variations in responses to experimental manipulations and introduces significant noise into a dataset that can mask the signal from groups of animals responding to experimental and vehicle injections. To circumvent this somewhat by removing the confounding problem of genetic diversity, animals housed for long term in our facilities were employed to serve as their own vehicle injection controls for comparisons to injections of foreign particles, which is an approach that has been employed previously (45, 48, 53). Sea urchins that were evaluated for responses to repeated injections of vehicle (n = 7) were subjected to subsequent injections of either *V. diazotrophicus* (n = 4) or zymosan A (n = 3) after 1 to 5 months (see methods; **Table S1**). Baseline and injury data were collected at the start of each experimental manipulation and the coelomocyte baseline results were used to standardize each treatment, as described above. Flow cytometry scatter plots illustrated that the coelomocyte populations in individual animals had different responses to challenge with foreign particles compared to vehicle (**Figure S5**). These differences highlighted the value of using the same animal for both experimental and control treatments. Therefore, fold changes in total coelomocytes and populations of different coelomocytes relative to baseline were compared between responses to foreign particles and responses to vehicle for individual animals (**Figures 5, 6**; **S6**). Changes in the populations of coelomocytes showed different trends in response to *V. diazotrophicus* compared to vehicle for the four sea urchins. SU-V1 had the largest fold increases among the sea urchins for all cell types in response to the first injection with *V. diazotrophicus* (**Figures 5A–D**; **S6A**). The small phagocytes and RSCs in SU-V1 showed the greatest fold increases after the second injection of *V. diazotrophicus* compared to baseline (**Figures 5C, D**). On the other hand, SU-V2 was essentially non-responsive and showed few fold changes in coelomocyte populations in response to *V. diazotrophicus* compared to vehicle control (**Figures 5E–H**; **S6B**). SU-V3 and SU-V4 showed intermediate responses to *V. diazotrophicus* with overall fold increases of ≤2 in most coelomocyte populations compared to vehicle (**Figures 5I–P**; **S6C, D**). The strongest responses by SU-V3 and SU-V4 occurred on day 6, which were delayed relative to SU-V1 that showed fold increases in most coelomocyte populations on day 3.

Sea urchins challenged with zymosan A (SU-Z1, SU-Z2, SU-Z3) were compared to their responses to vehicle (**Figures 6**; **S6E-G**). SU-Z1 and SU-Z3 showed fold decreases over time for total coelomocytes and large phagocytes in response to both zymosan A and vehicle compared to baseline while SU-Z2 showed a slight fold increase (**Figures 6A–F**). However, the response to zymosan A by the total coelomocytes and large phagocytes showed less of a fold decrease on day 6 compared to vehicle (**Figures 6A–F**). Fold changes in the RSCs and small phagocytes for all three animals generally decreased on day 1 and day 3 and increased on day 6 (**Figures 6G–L**). The fold changes in the RSCs were noteworthy because they were similar for the three animals except that the fold increase for SU-Z3 was delayed and evident on day 6 rather than day 3 (**Figure 6L**). Furthermore, the small phagocyte population in SU-Z3 showed a fold increase that was greater than the fold increases for SU-Z1 and SU-Z2 (**Figures 6G–I**). In general, while both zymosan A and vehicle induced fold decreases in total coelomocytes and large phagocytes, this decrease was not as prominent for the three sea urchins responding to zymosan A. Results also indicated fold increases in both RSCs and small phagocytes that corresponded with decreases in large phagocytes. The variations among the different coelomocyte populations in sea urchins responding to zymosan A illustrated the idiosyncratic complexity of these cells among animals (**Figures 6D–L**; **S6E-G**).

## Discussion

### Coelomocyte responses to injury and immune challenge are dynamic and variable

Sea urchin coelomocytes respond in a variety of ways to infection and injury (reviewed in (28, 55). Damage to the body wall that breaks the rigid test or skeleton in echinoids cannot be closed as in sea stars that contract their tissues around an injury to stop the bleeding of CF. Consequently, echinoids rely on clotting processes to prevent lethal losses of CF, which is based on both cellular and protein clots (56–58). This clotting process likely involves both types of large phagocytes which undergo reversible lamellipodial to filopodial changes to the cytoskeleton *in vitro* in response to changes in the medium (29–31, 41, 56). *In vivo*, these morphological changes can result in cellular clots [also termed aggregates (56, 59)], which may explain the decreases in the number of large phagocytes in response to the initial injury observed here. This decrease would be consistent with their involvement in cellular clotting and encapsulating large particles by forming syncytia (21, 60, 61). Furthermore, it is feasible that putative signals from tissue damage plus chemokines and cytokines such as the members of the TNF and TNFR superfamilies, the FADD superfamilies, the IL17 family, and the MIF family of genes (4, 62–64) activate and/or attract phagocytes and keep them at a site of injury to seal a wound that compromises the body wall.

There are substantial changes in the populations of large phagocytes, small phagocytes, and RSCs in response to injection with foreign particles. The large phagocytes function as the major phagocytic cells in sea urchins as demonstrated by phagocytosis of bacteria, foreign cells, and foreign particles in short term cultures (36, 65–67) and clearance functions *in vivo* [(7, 8) reviewed in (44, 46)]. The populations of small phagocytes and RSCs also increase in response to foreign particles and therefore may be involved in responses to this type of foreign contact *in vivo* (9, 11, 28, 49, 68). Small phagocytes show elevated expression of SpTransformer (SpTrf) proteins in response to challenge, which are secreted and capable of binding to pathogen-associated molecular patterns (PAMPs) and opsonizing bacteria to augment phagocytosis (9, 11, 36, 49, 68, 69). In the presence of PAMPs or danger-associated molecular patterns (DAMPs), RSCs exocytose the contents of their vesicles that includes echinochrome A (38), which may reduce the viability of microbes and yeast based on iron chelation (70, 71). RSCs accumulate around bacteria *in vitro* (72) and surround the edges of surface injuries or infections that appear as dark red or black bands *in vivo* (73–76) including injuries from tissue grafting (77).

Here we report increases in large phagocyte concentration in response to *V. diazotrophicus* and decreases in response to zymosan A. The significant differences in size between these two foreign particles may underlie the difference in the responses by these cells; efficient phagocytosis of many small *V. diazotrophicus* cells vs. phagocytosis of only a few large zymosan A particles per phagocyte. Hence, more large phagocytes are likely required to clear zymosan A (35), however, the variability in the response and the small sample size likely reduced the identification of significant changes in the several cell populations. It is worth noting that the results for large phagocytes reported here in response to *V. diazotrophicus* differ from those reported by Yui and Bayne (7) in response to injections of Gram-negative and Gram-positive bacteria. They describe a 10 fold decrease in large phagocytes 24 hours after injection of marine bacteria, however, the types and numbers of live marine bacteria injected into the CF were 10 fold more compared to the single species of heat-killed *V. diazotrophicus* employed here. These differences may be the basis for the response reported by Yui and Bayne (7) for bacterial clearance, which appears similar to the clearance of zymosan A reported here. The greater numbers of injected bacteria may have required more large phagocytes for clearance that decreased their concentration.

It has been suggested that large phagocytes move from peripheral tissues into the CF to replace cells that function in phagocytosis or encapsulation and are removed from the CF during particle clearance (11). Recruitment of large phagocytes into the CF may function to maintain an optimal proportion of these cells after they are used in response to injury through clot formation and particle clearance by phagocytosis or encapsulation. Consequently, the concept of maintaining optimal proportions of cell types may be related to some of our results that are not in concordance for concentration compared to proportion. This is best illustrated by animals that received vehicle and showed changes in the large phagocytes of decreased concentration but increased proportion. This is in stark contrast to the response to zymosan A where the large phagocytes show a decrease in both concentration and proportion, which may indicate that the recruitment of large phagocytes from the periphery was unable to maintain normal proportions of large phagocytes in the CF. The large phagocyte response to *V. diazotrophicus* is different from either of the other treatments and shows an increase in concentration of these cells and a slight decrease in proportion. For responses to injections of *V. diazotrophicus* or vehicle, cell recruitment may function to maintain a relatively large phagocyte proportion, but for the clearance of zymosan A the rate of replacement may not be evident in the time scale of this analysis. The number of available large phagocytes in the tissues that may be recruited into the CF is likely limited and the appearance of newly proliferated large phagocytes is slow; only about 10% over 20 days (11). The process of recruiting large phagocytes from the tissues is key to both clearing foreign particles and maintaining an optimal proportion of large phagocytes in the CF. Depending on the insult to the animal, the ability to maintain an optimal proportion of large phagocytes in the CF may be overwhelmed in some circumstances such as clearance of zymosan A or response to an infecting pathogen.

Responses to *V. diazotrophicus* increases the concentration of small phagocytes, and increases in both concentration and proportion of RSCs, whereas injections of vehicle has little effect on these cell populations. Zymosan A induces consistent but less robust increases in the concentrations and proportions of both the small phagocytes and RCSs, which is consistent and very similar in all sea urchins, whereas responses to *V. diazotrophicus* are variable among animals. These results may be indicative of the differences in the cellular immune response to bacteria compared to yeast, which may be attributed in part to the size of the particles. The efficiency of clearance of bacteria by the large phagocytes may reduce the requirement to recruit RSCs and small phagocytes into the CF. In contrast, the large size of zymosan A particles and the limited numbers that any one phagocyte can take up (35) may increase the clearance time and may involve more large phagocytes, which may result in increased recruitment of RSCs and small phagocytes.

### Individual sea urchins show highly variable responses to immune challenge

The genetic variability of 4-5% among individual *S. purpuratus* (54) combined with their larval vs. adult life histories impacts the types of microbes and pathogens with which they come into contact. In addition, housing and maintenance of sea urchins varies among laboratories and depends on the scope and type of research being conducted. In our research on sea urchin immunity, animals are often kept long-term in aquaria with re-circulating artificial seawater (9, 11, 33), while in other labs, sea urchins may be housed in water tables with seawater pumped from the open-ocean (50, 78–80) or are used for analysis soon after collection (7, 8, 15, 72, 81). Immunoquiescence is the outcome of maintaining sea urchins for long term in recirculating aquaria and away from natural seawater, which was deduced from the down-regulation of *SpC3* and *SpEchinoidin* expression (10, 33, 45) and decreased coelomocyte concentration in the CF (9). All of this may be integrated into the complexities and variations of the immune responses that are displayed among animals including variations in coelomocyte populations. These results suggest a high level of individual variation among sea urchins prior to experimental immune challenges. Reports on the immune responses in echinoids often describe coelomocyte populations as the average for a group of animals (7, 8, 28, 80) with little information on individual animals or mention of the variations among them. This can be further confounded by the number of animals analyzed in a group, which is typically small (11, 45, 53).

Here we present results from 11 animals for their individual responses to challenge with foreign particles compared to their responses to vehicle. Not only is there great variation in responses among these animals, but there is also variation in the timing of changes in coelomocyte populations. This variation may be driven by any number of factors such as the age and size of the animal (82), genetic variation among animals (54), the sex of the animal (83), or environmental stress (76, 84–87). Although these unknowns may have contributed to the variability in the cellular responses among sea urchins, our approach highlights the need to use each individual animal as its own control.

### The cellular immune response in echinoids

The diverse types of sea urchin coelomocytes that function in the CF likely have interdependent activities to coordinate and optimize responses to immune challenges and to maintain optimal homeostasis of the relative populations of these cells and the health physiology of the animals. To understand the cellular immune system in this animal, the responses of each coelomocyte population to the particular challenge as well as the interactions among the cell populations must be taken into consideration. The results presented here suggest a scenario for how the cellular immune response in sea urchins may function as a unit. In the timeline of the challenges conducted here, the first impact on the animal is a puncture injury that incurs an open wound through the peristomial membrane plus possible punctures or tears to the internal membrane that separates the pharyngeal and coelomic spaces. The decreased concentration of large phagocytes in the CF likely correlates with phagocyte migration to the site(s) of injury and induction of a clotting reaction to block loss of CF. However, these cells maintain a steady but slightly increasing proportion of large phagocytes in the CF. The phagocyte response to injury is transient and shows little to no changes in the populations of the other types of coelomocytes. If the injury is associated with injection of foreign particles into the CF, then a more active immune response is triggered. When foreign particles are either small or few in number, as for *Vibrio diazotrophicus*, the phagocytes respond to the injury as inferred from the initial decrease in concentration, but the cell numbers recover, likely through the recruitment of cells from the periphery into the CF to replace those used to clear the perceived pathogen. Although the large phagocyte concentration is variable over time in response to *V. diazotrophicus*, these cells maintain a relatively steady proportion of the coelomocytes during this clearance activity. When the foreign particle is large as for zymosan A, there is a sustained decrease in both the concentration and proportion of large phagocytes, which is different from clearance of *V. diazotrophicus*. These differences may be the outcome of different numbers of large phagocytes that are required to clear *V. diazotrophicus* vs. the larger zymosan A particles. Furthermore, encapsulation of aggregated particles may involve many more large phagocytes in a combination of a cellular clot and a protein clot that binds to and entangles cells [*e.g*., see (57)].

If the large phagocytes clear the foreign particles efficiently, then changes in the populations of the other coelomocytes are not observed, which appears to be the case for SU-V2 and SU-V3 responding to *V. diazotrophicus* (**Figure 5**). However, if the large phagocytes do not clear *V. diazotrophicus* quickly, as observed for SU-V1 and SU-V4 and for all sea urchins responding to zymosan A, then the populations of the small phagocytes and red spherule cells increase in both concentration and proportion (**Figures 5**, **6**). In general, the large phagocytes appear to be the primary responders for clearing foreign particles, and depending on the type of particle, require different numbers of phagocytes, which can take more or less time. Based on this response, the other cell types appear to be recruited when the large phagocytes do not respond efficiently and the population decreases as recorded within the time frame of this analysis.

## Conclusions

Past studies show that the coelomocytes in sea urchins change in the CF over time in response to immune challenges (7–9, 11–13, 15), but the results have not been reported based on quantitative analysis of the different types of coelomocytes. The results described here track specific populations of coelomocytes from individual sea urchins over the course of two types of immune challenge; Gram-negative marine bacteria, *V. diazotrophicus*, and zymosan A, a glucan prepared from the cell wall of the yeast, *Saccharomyces cerevisiae*. Results from both challenges compared to injections of vehicle differentiates between injury and responses to foreign particles. *Vibrio diazotrophicus* induces variable changes in coelomocyte types, depending on the individual sea urchin challenged, and shows an average increase in all coelomocyte types, whereas the response to zymosan A decreases the large phagocyte population and increases the small phagocytes and RSCs. These distinct differences in responses by sea urchin coelomocytes to *V. diazotrophicus* vs. zymosan A may be the outcome of the immuno-stimulatory differences between the Gram-negative bacteria and the fungal glucan, in addition to a single PAMP versus a complex mix of potential PAMPs presented by the *Vibrio* cells. Furthermore, the significant differences in particle sizes likely impacts the clearance parameters and success by the large phagocytes, which, in turn, impacts how each cell population changes. This infers a series of events in the cellular immune system of the sea urchins that are driven by the large phagocyte response based on their ability to clear foreign particles successfully from the CF. Variability in coelomocyte responses among individual sea urchins makes analyses difficult when employed as groups that ignores individual variation in response. This variation in response among sea urchins is likely an advantage to the population in their normal habitat in which variability may aid in species survival from constantly changing pathogens.

## Data availability statement

The raw data supporting the conclusions of this article will be made available by the authors, without undue reservation.

## Author contributions

MABH contributed to the conception and design of the study. LG provided guidance for flow cytometry. MABH, LG, and LCS contributed to writing and editing the manuscript. LCS and LG acquired funding to support this research. All authors contributed to the article and approved the submitted version.

## Funding

This research was supported by a US National Science Foundation award (IOS-1855747) to LCS and LG, and awards from the Wilbur V. Harlan Trust through the Department of Biological Sciences and the George Washington University Columbian College of Arts and Sciences to MABH.

## Acknowledgments

Dr. Damien O’Halloran provided helpful feedback on an early version of this manuscript. The authors are grateful for the help from Caroline R. Reynolds and Chloe G. Shaw for excellent animal care and aquarium maintenance.

## Conflict of interest

The authors declare that the research was conducted in the absence of any commercial or financial relationships that could be construed as a potential conflict of interest.

## Publisher’s note

All claims expressed in this article are solely those of the authors and do not necessarily represent those of their affiliated organizations, or those of the publisher, the editors and the reviewers. Any product that may be evaluated in this article, or claim that may be made by its manufacturer, is not guaranteed or endorsed by the publisher.
