# Supplementary material for: Coelomocyte populations in the sea urchin, Strongylocentrotus purpuratus, undergo dynamic changes in response to immune challenge
Source: Front Immunol. 2022 Aug 31;13:940852. doi: 10.3389/fimmu.2022.940852 (PMC9471872; doi:10.3389/fimmu.2022.940852)
Supplement: Supplementary file 1 [file DataSheet_1.pdf]

# Supplemental Files

# Coelomocyte populations in the sea urchin, *Strongylocentrotus purpuratus*, undergo dynamic changes in response to immune challenge

**Megan A. Barela Hudgell, Leon Grayfer, L. Courtney Smith**

Department of Biological Sciences, George Washington University, Washington, DC 20052, USA.

## Supplemental Figures

Figure S1 | Flow cytometry gates are used to analyze and quantify sea urchin coelomocyte populations.

Figure S2 | Injections of heat-killed *Vibrio diazotrophicus*, zymosan A, or vehicle (aCF) induce different fold changes in coelomocytes.

Figure S3 | Injections of heat-killed *Vibrio diazotrophicus*, zymosan A, or vehicle induce different fold changes in coelomocytes.

Figure S4 | The population of vibratile cells and colorless spherule cells (CSCs) do not show significant changes in response to injections of heat-killed *Vibrio diazotrophicus* or zymosan A compared to vehicle (aCF).

Figure S5 | Selected sea urchins injected with different foreign particles show differences in coelomocyte populations.

Figure S6 | The mixed population of vibratile cells and colorless spherule cells (CSCs) do not show significant changes in response to injections of heat-killed *Vibrio diazotrophicus* or zymosan A compared to vehicle (aCF).

Figure S7 | Fold changes in different coelomocyte types in response to injection with foreign particles is variable among sea urchins.

## Supplemental Table

Table S1 | Sea urchins used in this study

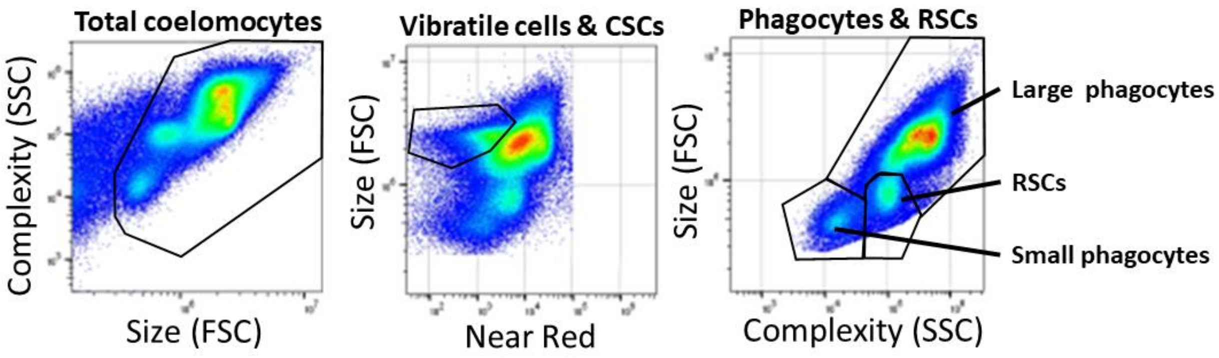


**Figure S1 | Flow cytometry gates are used to analyze and quantify sea urchin coelomocyte populations.** Dot plots from FlowJo were edited using Procreate and PowerPoint to produce three representative figures. These show the flow cytometry dot plots and gates used to identify total coelomocytes, vibratile cells and CSCs, large phagocytes, small phagocytes, and RSCs. Total coelomocytes are gated based on (1). Large phagocytes, small phagocytes, and RSCs are gated based on (2).

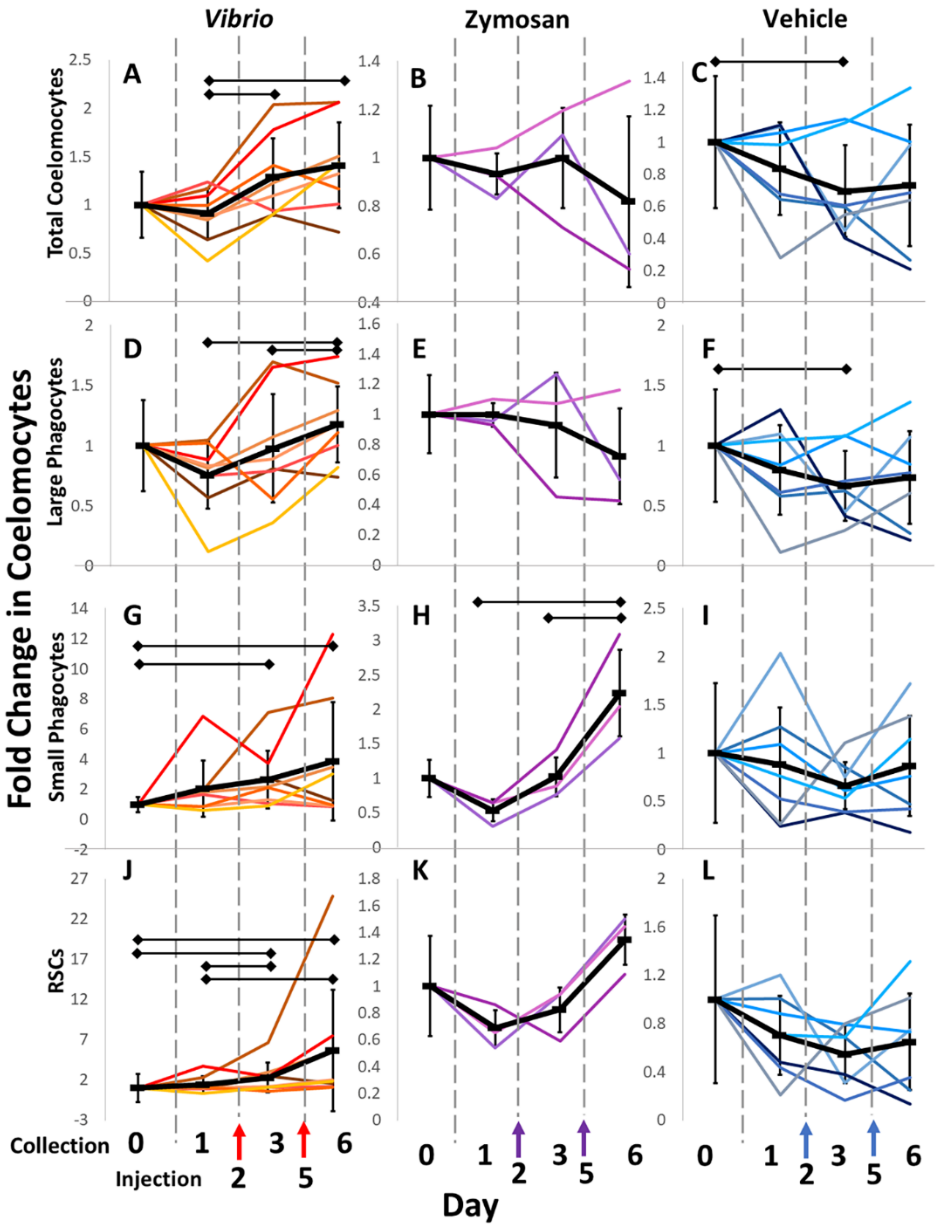


**Figure S2 | Injections of heat-killed *Vibrio diazotrophicus*, zymosan A, or vehicle (aCF) induce different fold changes in coelomocytes.** Coelomocytes from sea urchins injected twice on days 2 and 5 (indicated by colored arrows along the x-axis) with either *Vibrio diazotrophicus* (**A**, **D**, **G**, **J**; n = 8; red lines), zymosan A (**B**, **E**, **H**, **K**; n = 3; purple lines), or vehicle as the injury control (**C**, **F**, **I**, **L**; n = 7; blue lines) were evaluated on days 3 and 5. Responses to challenges are compared to baseline (day 0; set to 1) and to injury and CF withdrawal (day 1). Fold changes in coelomocyte populations relative to baseline for all sea urchins are shown for total coelomocytes (**A-C**), large phagocytes (**D- F**), small phagocytes (**G- I**), and red spherule cells (RSCs; **J-L**). See Figure S4A-C for fold changes in vibratile cells and colorless spherule cells (CSCs). Colored lines indicate responses over time for individual animals. Average ± SD are indicated for each time point for each experimental group. Black horizontal bars indicate significant differences (unpaired *t*-test: $p \leq 0.05$).

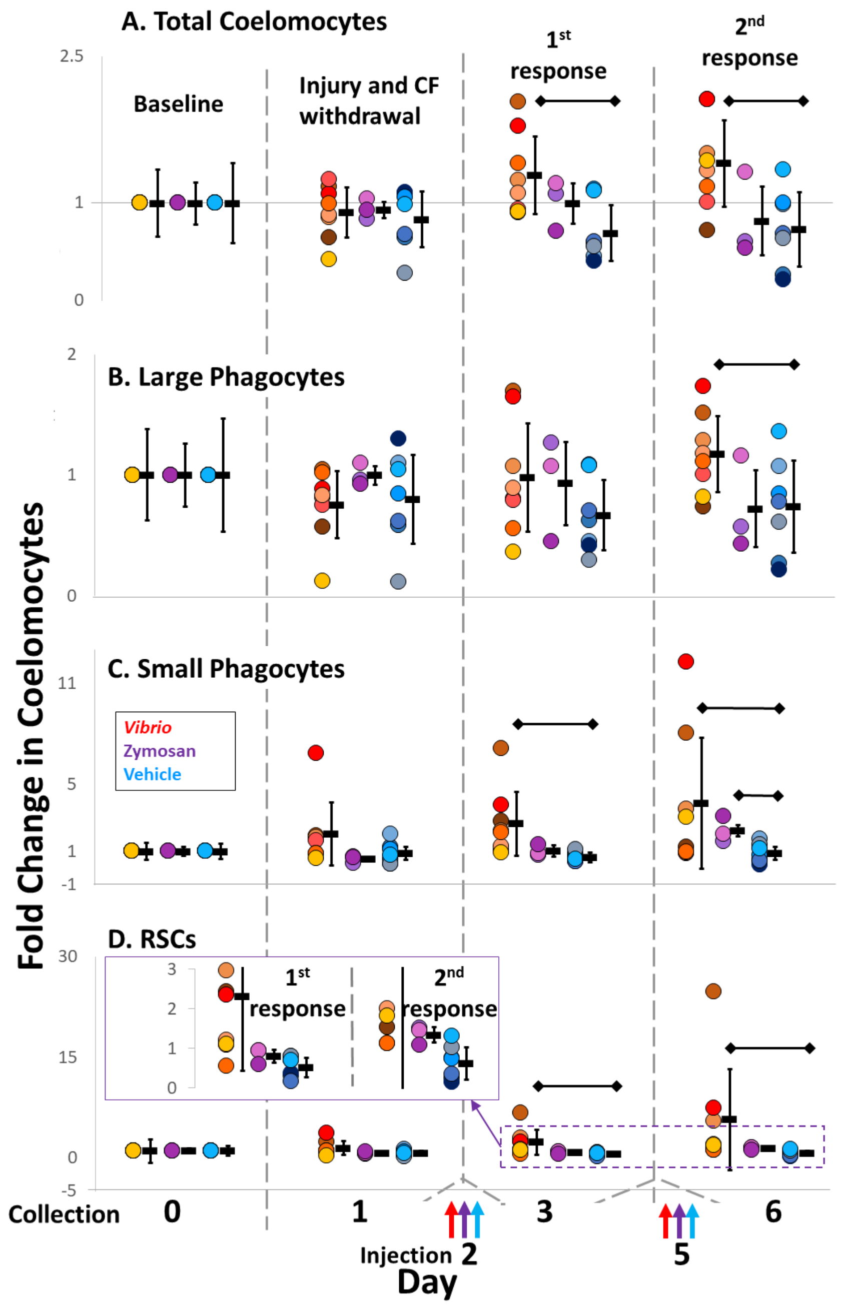


**Figure S3 | Injections of heat-killed *Vibrio diazotrophicus*, zymosan A, or vehicle induce different fold changes in coelomocytes.** Populations of large phagocytes (**A**), small phagocytes (**B**), and RSCs (**C**) from groups of sea urchins were evaluated prior to challenge at baseline (set to 1) and in response to injury and CF withdrawal. Responses to challenge with either *Vibrio diazotrophicus* or zymosan are compared to responses to vehicle. Dots indicate results for individual animals and the legend correlates treatment with color. Mean ± SD are located to the right of each set of dots. Black horizontal bars indicate significant differences between responses to foreign particles vs. vehicle (ANOVA, $p \leq 0.05$). Boxed dots in (**C**) are expanded in the inset. See Figure S4D for corresponding results for populations of the vibratile and CSCs in individual animals in response to challenge.

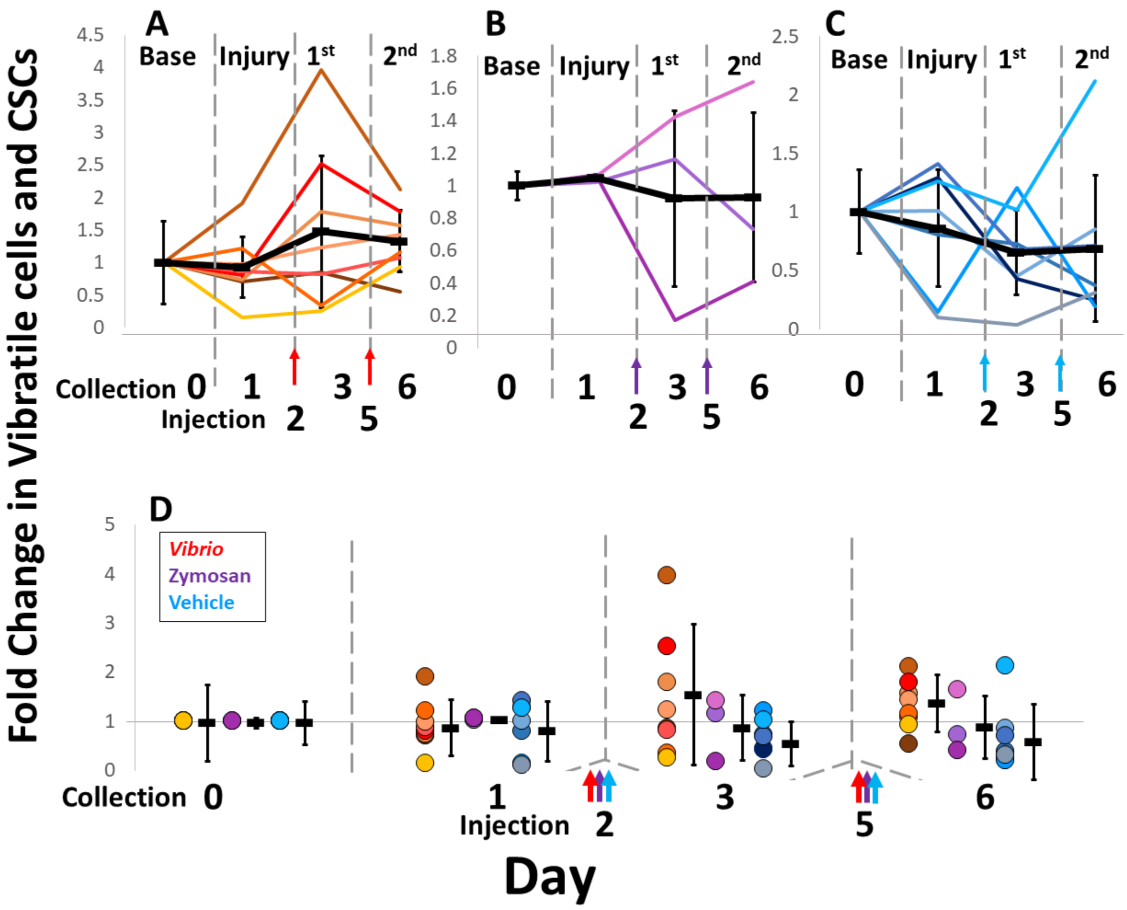


**Figure S4 | The population of vibratile cells and colorless spherule cells (CSCs) do not show significant changes in response to injections of heat-killed *Vibrio diazotrophicus* or zymosan A compared to vehicle (aCF).** Fold changes in populations of vibratile cells and CSCs were analyzed by flow cytometry and tracked over time. On days 2 and 5, sea urchins were injected (indicated with colored arrows at the x axes) with either *Vibrio diazotrophicus* (red arrows), zymosan A (purple arrows), or vehicle (blue arrows). Cell responses are shown as fold change compared to baseline (day 0, set to 1). (**A-C**) **Fold change over time in vibratile cells and CSCs.** Colored lines indicate fold changes in populations of vibratile and CSC populations in response to injury and CF withdrawal and injections of *Vibrio diazotrophicus* (**A**), zymosan A (**B**), or vehicle (**C**) in individual sea urchins. Black lines indicate the average response over time for all animals in each experimental group. No significant differences are identified in these cell populations over time (unpaired *t*-test; $p \leq 0.05$). Average fold change ± SD are indicated for each time point. (**D**) **Fold change in cell populations over time are compared among groups.** Injection of heat-killed *Vibrio diazotrophicus*, zymosan A, or vehicle do not induce significant fold changes in vibratile cells and colorless spherule cells (CSCs) in individual sea urchins. Fold changes in populations of vibratile and CSCs in response to challenge are compared to responses to vehicle. The legend correlates treatment to color. Each dot indicates results for an individual animal. Average ± SD are shown to the right of each set of dots. No significant changes in response to foreign particles vs. vehicle are identified for this cell population (ANOVA, $p \leq 0.05$).

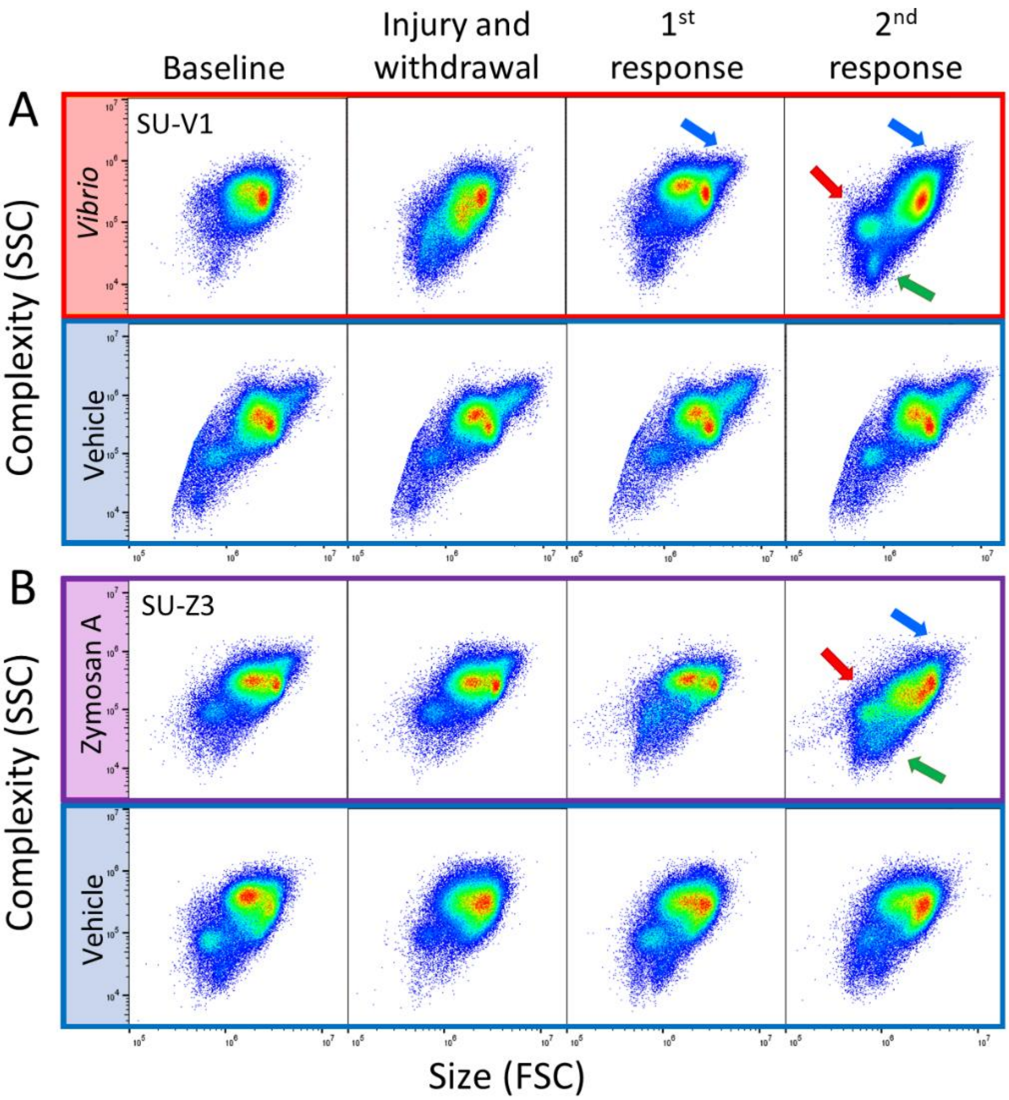


**Figure S5 | Selected sea urchins injected with different foreign particles show differences in coelomocyte populations.** Sea urchins that initially received injections of vehicle, were allowed to recover, and then were injected with either *Vibrio diazotrophicus* (SU-V1) or zymosan A (SUZ-3). Coelomocytes were evaluated by flow cytometry at baseline (day 0), for the response to injury and CF withdrawal (day 1), and for responses 24 hours after the first and second injections of foreign particles (days 3, 6). Specific coelomocytes populations are indicated by arrows: large phagocytes (blue arrows), RSCs (red arrows), and small phagocytes (green arrows). A variety of changes in coelomocyte populations were observed in response to injections of foreign particles compared to vehicle. In response to *V. diazotrophicus*, SU-V1 shows an increase in the populations of RSCs and small phagocytes, plus a shift in the scatter plot for the large phagocyte population that indicates increased internal cellular complexity. In response to zymosan A, SU-Z3 shows an increase in the populations of RSCs and small phagocytes in addition to a shift in the large phagocytes. In each case, responses to the immune stimuli are quite different from the responses to vehicle, which does not show overt changes in populations in response to the two injections of vehicle. In general, the plots show that the coelomocyte populations at baseline (day 0) are different for both sea urchins prior to injections and were also different over time for the

same animal at baseline and in response to foreign particles or to vehicle. These results illustrate the variations in coelomocyte populations among animals and the difficulties in establishing statistical significance for groups of sea urchins. The differences are likely due to the significant genetic diversity among sea urchins and the unknown immunological history of pathogen contact and stressor impacts from the environment and housing in aquaria that can influence the composition of coelomocytes. The variations in the results highlight the value of using the same animal for both experimental and control treatments.

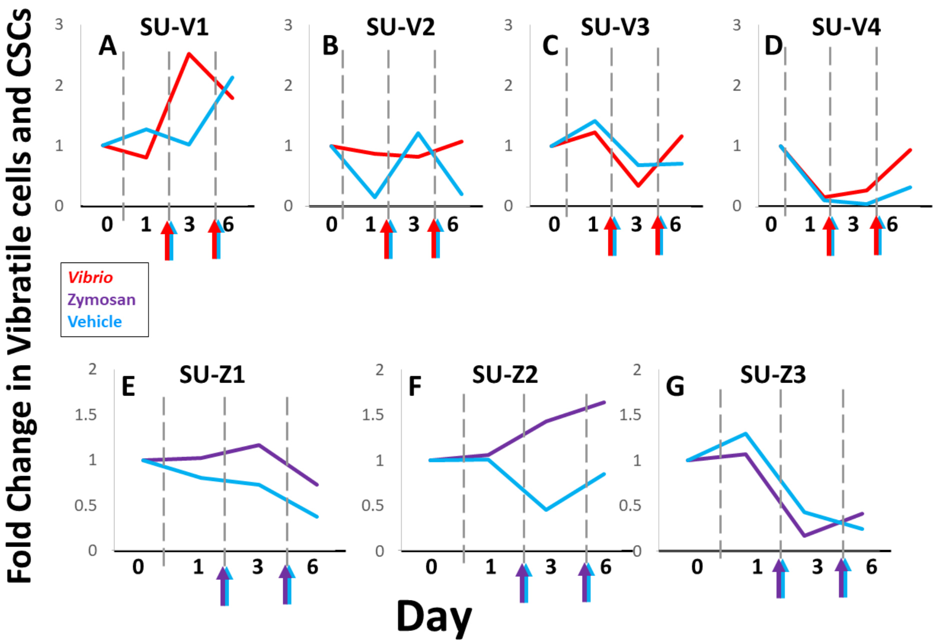


**Figure S6 | The mixed population of vibratile cells and colorless spherule cells (CSCs) do not show significant changes in response to injections of heat-killed *Vibrio diazotrophicus* or zymosan A compared to vehicle (aCF).** (**A-D**) **The mixed population of vibratile cells and CSCs from individual sea urchins (SU-V2, SU-V3, SU-V4) is variable in response to *Vibrio diazotrophicus*.** The population of vibratile cells and CSCs were evaluated initially after two injections of vehicle (blue lines). After recovery from the control injection, sea urchins were injected twice with *V. diazotrophicus* (red lines), and the mixed population of vibratile cells and CSCs was evaluated again. Colored arrows at the x axes indicate days 2 and 5 when sea urchins were injected with either *Vibrio diazotrophicus* (red arrows) or vehicle (blue arrows). SU-V1 was treated in the opposite order; it received *V. diazotrophicus* first followed by vehicle. All data points for each sea urchin are standardized to their individual baseline (day 0, set to 1). Fold changes in coelomocytes are compared across days for each animal. The mixed population of vibratile cells and CSCs from sea urchins challenged with *V. diazotrophicus* showed variable responses compared to the same sea urchins challenged with vehicle. Changes in the population was not similar among the four sea urchins, with no discernable pattern in responses to either *V. diazotrophicus* or to vehicle. (**E-G**) **The mixed population of vibratile cells and CSCs is variable among individual sea urchins (n = 3; SU-Z1, SU-Z2, SU-Z3) responding to injections of zymosan A.** Cells were evaluated initially after two injections of vehicle (blue lines). After a month of recovery from the control injection, sea urchins were injected twice with zymosan A (purple lines) and cells were evaluated again. Colored arrows at the x axes indicate days 2 and 5 when sea urchins were injected with either zymosan A (purple arrows) or vehicle (blue arrows). All points are standardized to their individual baseline (day 0, set to 1). Fold changes in coelomocytes are compared across days for each animal. The mixed population of vibratile cells and CSCs show variable responses to zymosan A with no discernable pattern in the responses among the animals. Overall, these results may be complicated by the failure to separate these two cell types for separate analysis, and changes in one may obscure changes in the other. Furthermore, these two cell types may have different functions than the other types of coelomocytes and may not respond to the challenges evaluated here.

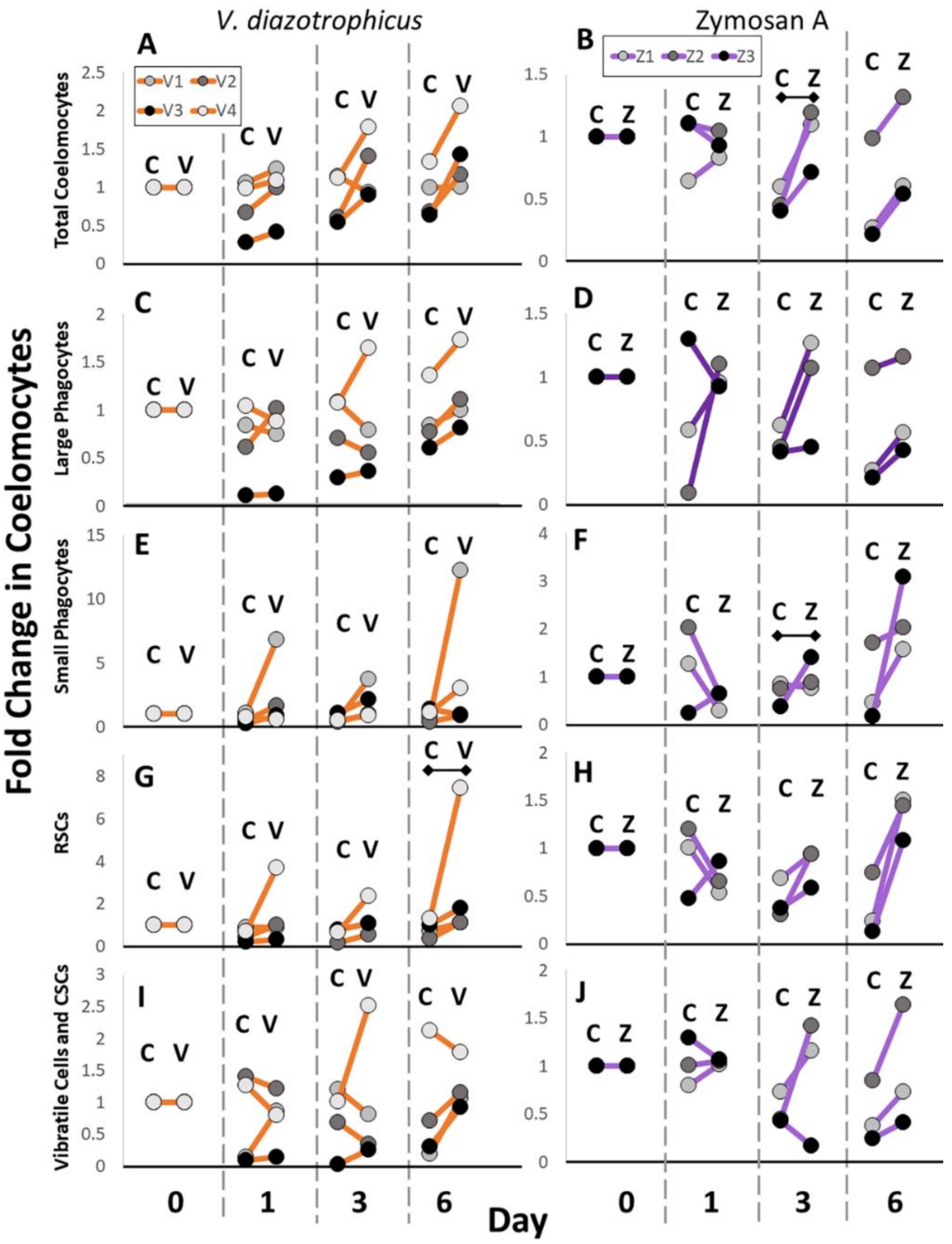


**Figure S7 | Fold changes in different coelomocyte types in response to injection with foreign particles is variable among sea urchins**. Because a statistical analysis cannot be conducted on single data points, the data from individual sea urchins that were used as their own control was re-evaluated as groups to conduct paired *t*-tests to determine whether the response to foreign particles was significantly different for each sea urchin responding to vehicle. Sea urchins (n = 7) were evaluated for their responses to vehicle (control; c), and compared to their responses to either *Vibrio diazotrophicus* (v; n = 4) or zymosan A (z; n = 3) (see methods in the main paper). Results for each sea urchin is standardized to their individual baseline (day 0, set to 1). Fold changes are shown for total coelomocytes (**A**, **B**), and populations of large phagocytes (**C**, **D**), small phagocytes (**E**, **F**), red spherule cells (RSCs; **G**, **H**) and the mixed population of vibratile cells and colorless spherule cells (CSCs; **I**, **J**) responding to injections with foreign particles (v or z) compared to fold

change in response to vehicle (c). See the legends that define line color with challenge and dot shading with individual sea urchins. Black horizontal bars indicate significant differences (paired *t*-test; $p \leq 0.05$). In general, most responses to injection with *V. diazotrophicus* (n = 4) or zymosan A (n = 3) do not show statistically significant fold changes for either total coelomocytes or for different coelomocyte populations compared to responses to vehicle. A few exceptions include significant fold increases in total coelomocytes ($p = 0.02$) and small phagocytes ($p = 0.05$) for sea urchins responding to zymosan A on day 3. RSCs show a significant fold increase ($p = 0.02$) in response to *V. diazotrophicus* compared to vehicle. Although most of these results are not significant, a degree of useful information can be gleaned. For example, three of four animals responding to *V. diazotrophicus* show greater fold increases in total coelomocytes, small phagocytes, and vibratile and CSCs compared to responses to vehicle. All seven sea urchins show greater fold increases in large phagocytes and RSCs in response to both injected particles compared to responses to vehicle. The three sea urchins injected with zymosan A have greater fold increases in all coelomocyte types compared to vehicle. Overall, increased fold changes in cell populations responding to both foreign particles relative to vehicle are observed in 61 of 70 comparisons, highlighting the value of employing individual sea urchins as their own controls, particularly when significant changes are not evident.

## Supplemental Table

**Table S1 |** Sea urchins used in this study

<table>
<tr><th></th><th></th><th colspan="3">Injected with</th></tr>
<tr><th>Treatment groups</th><th>n</th><th>Vibrio</th><th>Zymosan A</th><th>Vehicle[1]</th></tr>
<tr><td>Animals that received Vibrio</td><td>4</td><td rowspan="2">8</td><td rowspan="2"></td><td></td></tr>
<tr><td>Animals that received Vibrio and vehicle on separate dates</td><td>4</td><td rowspan="2">7</td></tr>
<tr><td>Animals that received zymosan A and vehicle on separate dates</td><td>3</td><td></td><td>3</td></tr>
<tr><td>Sea urchins used in this study</td><td>11</td><td></td><td></td><td></td></tr>
<tr><td>Samples collected in this study</td><td>18</td><td></td><td></td><td></td></tr>
</table>

[1]Vehicle is aCF.

## References


1. Smith LC, Hawley TS, Henson JH, Majeske AJ, Oren M, Rosental B. Methods for collection, handling, and analysis of sea urchin coelomocytes. In: Foltz KA, Hamdoun A, editors. Echinoderms Methods in Cell Biology. Vol. 150A. 2019. p. 357–89 doi:10.1016/bs.mcb.2018.11.009.

2. Barela Hudgell MA, Grayfer L, Smith LC. A flow cytometry based approach to identify distinct coelomocyte subsets of the purple sea urchin, *Strongylocentrotus purpuratus*. *Dev Comp Immunol*. (2022) 130:104352. doi:10.1016/j.dci.2022.104352.
